# Planktonic and Biofilm-Associated *Pseudomonas aeruginosa* and *Staphylococcus epidermidis* Elicit Differential Human Peripheral Blood Cell Responses

**DOI:** 10.3390/microorganisms9091846

**Published:** 2021-08-31

**Authors:** Esingül Kaya, Giovanna Batoni, Mariagrazia Di Luca, Eleonora Apolloni, Alessandro Mazzoni, Giuseppantonio Maisetta, Semih Esin

**Affiliations:** 1 Department of Translational Research and New Technologies in Medicine and Surgery, University of Pisa, 56123 Pisa, Italy; e.kaya@studenti.unipi.it (E.K.); giovanna.batoni@unipi.it (G.B.); e.apolloni@studenti.unipi.it (E.A.); giuseppantonio.maisetta@unipi.it (G.M.); 2 Department of Biology, University of Pisa, 56123 Pisa, Italy; mariagrazia.diluca@unipi.it; 3 Department of Transfusion Medicine and Transplant Biology, Pisa University Hospital, 56124 Pisa, Italy; a.mazzoni@ao-pisa.toscana.it

**Keywords:** biofilm, immune response, planktonic cells, peripheral blood mononuclear cells, *Pseudomonas aeruginosa*, *Staphylococcus epidermidis*

## Abstract

Despite the considerable progress made in recent years, our understanding of the human immune response to microbial biofilms is still poor. The aim of the present study was to compare the in vitro response of human peripheral blood mononuclear cells (PBMC) to biofilms and planktonic cells of *Pseudomonas aeruginosa* and *Staphylococcus epidermidis,* two bacterial species particularly relevant in patients with cystic fibrosis or undergoing endovascular catheterization, respectively. PBMC isolated from healthy donors were co-cultured with 24 h-old biofilms or with exponentially growing cells of both species. Following 24 h of co-culture, the expression of early activation markers and the levels of cytokines in the culture supernatants were assessed by flow cytometry, while biofilm biomass and architecture were evaluated by crystal violet staining, CFU count, and confocal microscopy. Around 20% of PBMC was activated in response to both biofilms and planktonic cells of *P. aeruginosa*. In contrast, planktonic cells of *S. epidermidis* induced a statistically higher degree of activation than their biofilm counterpart (25% versus 15%; *p* < 0.01). *P. aeruginosa* biofilms stimulated pro-inflammatory (TNF-α, IL-1β, IFN-γ, and IL-6) and anti-inflammatory (IL-10) cytokine production at statistically significant levels higher than its planktonic counterpart, while an opposite trend was observed with *S. epidermidis*. Differences in the architecture of the biofilms and in the number of PBMC infiltrating the biofilms between the two bacterial species may at least partially explain these findings. Collectively, the results obtained highlighted marked differences in the host–cell response depending on the species and the mode of growth (biofilms versus planktonic cultures), allowing speculations on the different strategies adopted by *P. aeruginosa* and *S. epidermidis* to persist in the host during the course of chronic infections.

## 1. Introduction

Biofilms are complex bacterial communities encased within an extracellular polymeric substance (EPS) [1]. They have the ability to develop on both host native tissues and artificial surfaces [1]. These latter include a wide variety of implanted medical devices (e.g., indwelling catheters, artificial heart valves, orthopedic prostheses, and dental implants), which are essential tools in modern clinical practice [2]. Due to an intrinsic tolerance of biofilm-embedded bacteria to antimicrobial treatment and clearance by the host immune system, the management of biofilm-associated infections is particularly challenging [3,4]. The biofilm removal from the host tissues through invasive surgical procedures or the explantation of the medical device are sometimes the only therapeutic options [3]. Such procedures lead to an increase in the rate of hospitalization and health care costs and lower patients’ quality of life [3]. In such circumstances, biofilms are prone to recurrence, constituting a chronic stimulus for the host immune response, which is considered largely ineffective in biofilm removal [5]. 

The developmental stages leading to biofilm formation seem to be conserved across different species and include an adhesion phase, a maturation phase and a dispersal phase [6]. Nevertheless, every species forms a unique multicellular community with marked differences in architecture, chemical composition, or intercellular communication systems (e.g., quorum sensing signals) among mature biofilms of different microbial species, including strains within a species [6]. For instance, in most experimental conditions and in minimal glucose media *Pseudomonas aeruginosa*, one of the most frequent pathogens isolated from patients with chronic infections, such as chronic wounds and cystic fibrosis (CF), typically forms multicellular structures with mushroom-shaped or tower-shaped microcolonies [7]. At least three different EPS exopolysaccharides, designated alginate, Pel, and Psl, are essential for the stability of the structure of *P. aeruginosa* biofilms [8]. The genetic capacity to produce such exopolysaccharides varies between strains and experimental conditions, with Pel and Psl being mainly produced in vitro and by the early colonizers of CF lung (non-mucoid isolates), while the alginate is often over-produced by the so-called mucoid strains that emerge in the CF lung as the infection becomes chronic [8]. The opportunistic pathogen *Staphylococcus epidermidis* is another strong biofilm producer responsible for the majority of nosocomial catheter-related blood stream infections [9,10]. It typically forms strongly adhesive and compact biofilms with an abundant EPS constituted by the polysaccharide intercellular adhesin (PIA), but PIA-negative biofilm producer strains have been described as well [9,10].

In addition to species/strain-dependent differences among pathogenic biofilms, several features distinguish biofilms from planktonic cells within the same species. These include rate of growth, metabolic activity, tolerance to environmental stresses, nutrient availability, chemical composition as well as global gene expression that has been demonstrated to considerably differ between planktonic cultures and their corresponding biofilms [11,12,13]. 

The increasing frequency of biofilm infections and their extraordinary resistance to antibiotics highlights the importance of understanding the host immune response to biofilms, in the hope of developing novel (immuno)-therapeutic approaches to manage biofilm-associated infections. Despite the progress made in this field over the past decade, we are only beginning to understand the multifaceted immune response against microbial biofilms and the unique features, if any, that characterize such response against different bacterial species or as compared to the planktonic counterparts [14].

In a previous study, we assessed the interactions of isolated human peripheral blood mononuclear cells (PBMC) with biofilms of *P. aeruginosa* [15]. A reciprocal interaction was demonstrated in which PBMC underwent strong activation and produced high amounts of pro-inflammatory cytokines upon co-culture with *P. aeruginosa* biofilms. Conversely, the load of biofilm-associated *P. aeruginosa* was markedly increased in the presence of PBMC and PBMC-derived components, suggesting ability of the bacterium to exploit the presence of immune cells to promote its persistency.

In this study, we take a step further by comparing the immune responses induced in vitro upon co-culture of human PBMC with planktonic cells and biofilms of *P. aeruginosa* and *S. epidermidis*. Collectively, the results obtained highlight important differences in the host-cell activation pattern and inflammatory response, depending on the bacteria species and the mode of growth (biofilms *versus* planktonic cultures). A discussion on the possible implications of such findings in the pathogenesis of *P. aeruginosa* and *S. epidermidis* biofilm-associated infections is provided.

## 2. Materials and Methods

### 2.1. Bacterial Strains and Culture Conditions 

Three reference strains used in this study were *Pseudomonas aeruginosa* ATCC 27853, *P. aeruginosa* PAO1, and *Staphylococcus epidermidis* ATCC 35984. In the case of *P. aeruginosa*, the results obtained with the two different strains (PAO1, 3 experiments) and ATCC27853 (4 experiments) were similar; therefore, the data obtained for these two strains were pooled throughout the manuscript. For the preparation of stock cultures, the strains were grown at 37 °C with shaking (150 rpm) in Tryptone Soy Broth (TSB) (Oxoid, Basingstoke, UK). After approximately 2 h, mid-exponentially phase cultures were aliquoted and stored at −80 °C until use. At the time of experiment, 50 µL from a thawed aliquote was inoculated in 5 mL of TSB. Following 18 h of incubation at 37 °C in shaking condition, the obtained stationary phase cultures were used as inocula for the preparation of the biofilms (see below). To obtain exponentially growing bacteria, 50 µL was taken from stationary phase cultures and inoculated in 5 mL of cell-culture medium consisting of RPMI 1640 (Euroclone S.p.A, Pero, Milan, Italy) supplemented with 10% heat-inactivated fetal bovine serum (Merck, non-USA origin, Milan, Italy) and 2 mM L-glutamine (Euroclone). Cultures were incubated at 37 °C with shaking (150 rpm) for approximately 2 h to let bacteria reach the mid-exponential growth phase. Colony-forming units (CFUs) were determined by plating serially diluted bacterial suspensions on Tryptone Soy Agar (TSA) (Oxoid). Colonies were counted after incubation of the plates at 37 °C for 24–48 h.

### 2.2. Biofilm Formation

An aliquot of 500 µL from the stationary phase cultures was washed by centrifugation at 4000× *g* for 5 min at room temperature. The supernatant was discarded, and the bacterial pellet was resuspended in 500 µL of RPMI 1640 (Euroclone), supplemented with 10% heat-inactivated fetal bovine serum (Merck, Milan, Italy) and 2 mM L-glutamine (Euroclone). Bacterial suspensions containing approximately 1 × 10^6^ CFU per well were inoculated into 96-well flat bottom plates (Euroclone) in a volume of 100 µL for *P. aeruginosa* and 50 µL for *S. epidermidis*. The 96-well plates were incubated at 37 °C for 24 h, without shaking, to allow biofilm formation. Biofilms were then gently subjected to three washes with phosphate buffered saline (PBS) to remove non-embedded bacteria and incubated for further 24 h with or without PBMC (see below) in complete RPMI in humidified air containing 5% CO_2_. 

Preliminary experiments were performed to evaluate the bacterial load in 24 h-old biofilms of *P. aeruginosa* and *S. epidermidis* in the adopted experimental conditions. To this aim, biofilms were mechanically detached from the wells by scraping for 60 s with a pipette tip. Bacterial suspensions were vigorously vortexed and plated in serial dilutions on TSA (Oxoid) for CFU count. The same procedure was followed to establish the viable count associated with 48 h-old biofilms. 

### 2.3. Biofilm Mass Quantification

A standard crystal violet (CV) staining assay was used to quantify biofilm biomass as previously described [16]. To this aim, 24 h-old biofilms were rinsed three times with PBS, dried for 1 h at 60 °C and incubated for 15 min with 1% (*w/v*) CV (bioMérieux, Florence, Italy). Following incubation, unbound CV was removed by extensively washing the plate with PBS. After drying the plates at 37 °C for 30 min, biofilm-associated CV was extracted with 33% acid acetic (Sigma Aldrich) and quantified by measuring the optical density at 570 nm (OD_570_) in a microplate reader (Multiskan FC, Thermo-Fisher Scientific, Monza, Italy).

### 2.4. PBMC Separation

Blood was drawn from donors attending the Transfusion center of Pisa University Hospital or from healthy volunteers after an informed consent was obtained. The study was conducted in accordance with the Declaration of Helsinki, and the protocol was approved by the local Ethical Committee (Comitato Etico Area Vasta Nord-Ovest, CEAVNO, Protocol 34743, 28 June 2018). 

PBMC were isolated from buffy coats by standard gradient separation as described previously [15]. Briefly, buffy coats were diluted 1:1 with PBS, 10% sodium citrate (*v/v*). Cell suspensions were layered on a density gradient (Lymphoprep, Cedarlane, ON, Canada), and subjected to 20 min centrifugation at 160× *g* at RT. Afterward, platelets in the supernatant were gently removed without disturbing the mononuclear layer at the interface. After a further centrifugation at 800× *g* for 20 min, PBMC were collected from the interface and washed three times in PRMI. Finally, PBMC were resuspended in complete RPMI, replacing the fetal bovine serum with 10% heat-inactivated autologous plasma.

### 2.5. Co-Culture of PBMC with Intact Biofilms, Disrupted Biofilms or Planktonic Bacteria 

Mature biofilms (24 h-old) of *P. aeruginosa* or *S. epidermidis* were gently rinsed three times with PBS to remove non-embedded bacteria. A volume of 200 µL of PBMC suspension (2 × 10^6^ cells/mL, 4 × 10^5^ PBMC/well) was added to intact biofilms (IB). Biofilms from parallel wells were disrupted, as described before (Section 2.2), to obtain suspensions of biofilm cells and resuspended in 100 µL of complete RPMI, which were referred to as disrupted biofilm (DB). DB were used as additional stimulants by incubating them with 100 µL of PBMC suspension (4 × 10^6^ PBMC/mL, 4 × 10^5^ PBMC/well) (Supplementary Scheme S1). In parallel, planktonic, exponentially growing bacteria were obtained as described in Section 2.1. Based on the OD value, the bacterial suspension was diluted in complete RPMI to obtain a number of planktonic bacteria in 100 µL of complete RPMI, corresponding to the number of 24 h-old biofilm-associated bacteria, determined in preliminary experiments (Supplementary Scheme S1). A volume of 100 µL of PBMC suspension (4 × 10^6^ PBMC/mL, 4 × 10^5^ PBMC/well) was added, and this experimental condition was referred to as planktonic bacteria (PC). PBMC alone were used as a negative control. PBMC:bacteria co-cultures were incubated at 37 °C in 5.5% CO_2_ for 24 h. Wells containing biofilms without PBMC were also established for bacterial count. 

Following incubation, PBMC suspensions, obtained from six wells of each experimental condition, were pooled. Cell viability in each experimental condition was evaluated taking a 50 µL aliquot from each of the pooled sample (see Section 2.6). Afterward, cell suspensions were centrifuged at 500× *g* for 5 min. Supernatants from each condition were sterile filtered (0.22 µm), aliquoted, and stored at −80 °C for further assays (i.e., cytokine determination). The pellet containing PBMC was resuspended in PBS and subjected to cell surface staining (see Section 2.7).

### 2.6. Evaluation of Cell Viability by Trypan Blue Dye Exclusion Test

Viability of PBMC was assessed by the trypan blue dye exclusion test following 24 h co-culture with planktonic or sessile *P. aeruginosa*/*S. epidermidis* or incubated alone as previously described [15]. To this aim, PBMC were diluted 5 times with 0.4% trypan blue. An aliquot of the suspension was inserted into a Burker counting chamber (Merck, Milan, Italy) and observed under 400× magnification with light microscope (Olympus CH20BIMF200, Olympus Italy, Segrate, Milan, Italy). Two operators independently counted alive (clear) and dead (blue) cell numbers from six different fields. The values obtained were averaged.

### 2.7. Immunofluorescence Staining for Cell Surface and Activation Markers

PBMC co-cultured with bacteria (IB, DB and PC) or incubated alone were stained by incubating them with saturating amounts of monoclonal antibodies (MAbs) directed against cell surface or activation markers for 30 min at 4 °C. Two- or three-color immunofluorescence staining was performed as previously described [17]. The following MAbs were used for the staining: fluorescein isothiocyanate (FITC)-conjugated anti-CD3 or anti HLA-DR; PE-conjugated anti-CD14, anti-CD19, or anti-CD69 (Miltenyi Biotec, Bergisch Gladbach, Germany); rhodamine-PE-cyanin 5.1(PC5)-conjugated anti-CD56 MAb (Beckman Coulter srl, Milan, Italy). Isotype matched mouse immunoglobulin G (IgG) Mabs (Miltenyi Biotec) were used as negative controls. Following the staining, cells were washed with PBS and fixed with 1% paraformaldehyde (Merck, Milan, Italy) in PBS for 24 h at 4 °C. Following incubation, cells were washed in PBS and analyzed by flow cytometry. At least 50,000 events were acquired ungated in a BD Accuri C6 flow cytometer (BD Biosciences, San Jose, CA, USA). BD Accuri C6 software (BD Biosciences) was used for computer-assisted analyses. For the analyses, a widely set gate was chosen in order to select the cells of interest on a two-parameter plot of side scatter (SSC) vs. forward-angle scatter (FSC) and excluding debris and irrelevant events; among these cells the percentage and mean fluorescence intensity (MFI) of the surface markers were calculated according to following panel combinations: negative control-FITC/-PE/-PC5; CD3-FITC/CD69-PE/CD56-PC5; HLA-DR-FITC/CD19-PE.

### 2.8. Quantification of Cytokines in Culture Supernatants

Six cytokines (IL-1β, IL-6, IL-10, IL-8, IFN-γ, and TNF-α) were quantified in the co-culture supernatants by a flow cytometer based multibead capture assay (LEGENDplex^TM^ Multi-Analyte Flow Assay Kit, BioLegend Inc., San Diego, CA, USA) following manufacturer’s instructions. Sensitivities of the assay were as follows: IL-1β, 0.65 ± 0.47 pg/mL; IL-6, 0.97 ± 1.46 pg/mL; IL-8, 1.90 ± 0.65 pg/mL; IL-10, 0.77 ± 1.18 pg/mL; IFN-γ 0.76 ± 0.53, pg/mL; TNF-α, 0.88 ± 0.27 pg/mL. Acquisition of the samples was performed with a BD Accuri C6 flow cytometer (BD Biosciences). Data were analyzed with the LegendPlex v8.0 Software (BioLegend Inc., San Diego, CA, USA), and amount of the cytokines were calculated based on a standard curve. Results were expressed as pg/mL or ng/mL, depending on the cytokine.

### 2.9. Characterization of P. aeruginosa and S. epidermidis Biofilms in the Adopted Co-Culture Conditions

The architecture of *P. aeruginosa and S. epidermidis* biofilms in the adopted co-culture conditions was analyzed by Confocal Laser Scanning Microscopy (CLSM). To this aim, *P. aeruginosa* and *S. epidermidis* re-suspended in complete RPMI were seeded in 8 well ibiTreat polymer coverslips (ibidi GmbH, Gräfelfing, Germany) as previously described [15,18]. After 24 h and 48 h of incubation, biofilms were gently washed with sterile MilliQ water (MerckMillipore, Darmstadt, Germany)) and stained with Syto9 (green) and propidium iodide (red), according to the protocol of Filmtracer^TM^ LIVE/DEAD^TM^ Biofilm Viability Kit (Thermo Fisher Scientific, Monza, Italy). A TCS SP5 II (Leica Microsystems Srl, Buccinasco, Milan, Italy) confocal microscope was used to observe the samples, using a 60 × 1.25 NA water immersion objective. Different samples for each condition were scanned. A length of 10 µm Axial stacks in the Z plane with a slice thickness of 1 µm were taken. In parallel wells, the total biofilm biomass was evaluated by crystal violet (CV) staining as described above (Section 2.3). The biofilm-associated viable bacteria count was also determined as described in Section 2.2.

In further experiments, 24 h-old *P. aeruginosa* or *S. epidermidis* biofilms were labeled with 2 × 10^−6^ M green fluorescent lipophilic dye PKH67 (Merck) according to the manufacturer’s instructions, washed, and PBMC (2 × 10^6^ cells/mL, 4 × 10^5^ PBMC/well) prelabeled with 2 × 10^−6^ M red lipophilic dye PKH26 (Merck) were added onto the wells as described in Section 2.5. Immediately after the addition of the cells, a time-lapse live imaging (9 plane confocal images at 63× magnification for each biofilm, every 5 min, for up to 150 min. at 37 °C with 5.5% CO_2_ atmosphere) of the co-cultures was performed by using Operetta CLS High-Content Analysis System (PerkinElmer Inc., Boston, MA, USA). Images were then analyzed by Harmony software (Perkin Elmer Inc., Boston, MA, USA).

### 2.10. Statistical Analysis

GraphPad In Stat (GraphPad Software, La Jolla, CA, USA) was used to evaluate the statistical significance of the data. Student’s t test for paired samples or non-parametric Wilcoxon signed-rank test were used. For multiple comparisons, parametric and non-parametric ANOVA for matched samples followed by Bonferroni or Dunn’s multiple comparisons tests, respectively, were used. A *p* value of < 0.05 was considered significant.

## 3. Results

### 3.1. Bacterial Cell Number at Time 0 and PBMC Vitality after 24 h Co-Culture with Bacteria

**Figure 1:**
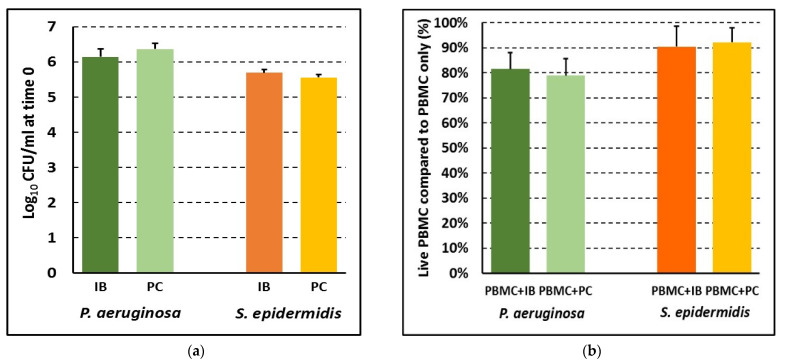
Number of planktonic (PC) and biofilm-associated bacteria (IB) at time 0 of co-culture with PBMC (**a**) and the percent of live PBMC co-cultured with intact biofilm (PBMC + IB) or planktonic bacteria (PBMC + PC) for 24 h with respect to PBMC incubated in the absence of bacteria (**b**). Mean values ± SEM of *n* = 7 (*P. aeruginosa*) and *n* = 8 (*S. epidermidis*) independent experiments are shown. *p* > 0.05 for both bacteria species. Student’s t test for paired samples.

In order to compare the PBMC-response to biofilms and planktonic bacteria within the same species, we first ensured that the bacterial loads in the two modes of growth were similar at time 0 and that such bacterial loads could warrant acceptable levels of PBMC vitality after 24 h of bacteria:cell co-culture. To this aim, in each experiment, 24 h-old biofilms of both species were scraped from several wells, and the CFU numbers were assessed after vigorous mixing. In parallel, CFU numbers were estimated in aliquots of exponential phase cultures growing in RPMI, after adjusting the OD to the cell density similar to that of biofilm-associated bacteria. As shown in Figure 1a, comparable numbers of biofilm-associated bacteria and planktonic bacteria were obtained for both *P. aeruginosa* and *S. epidermidis*, suggesting that the overall bacterial stimulus in the two conditions was similar at time 0 of co-culture with host cells. As compared to PBMC incubated without bacteria, after 24 h of co-culture with *P. aeruginosa*, approximately 80% of PBMC were alive while the percentage reached 90% in the case of *S. epidermidis*, with no statistically significant difference between biofilms and planktonic bacteria for both species (Figure 1b). 

### 3.2. Activation of PBMC Stimulated with P. aeruginosa or S. epidermidis in Planktonic or Biofilm Mode of Growth

**Figure 2:**
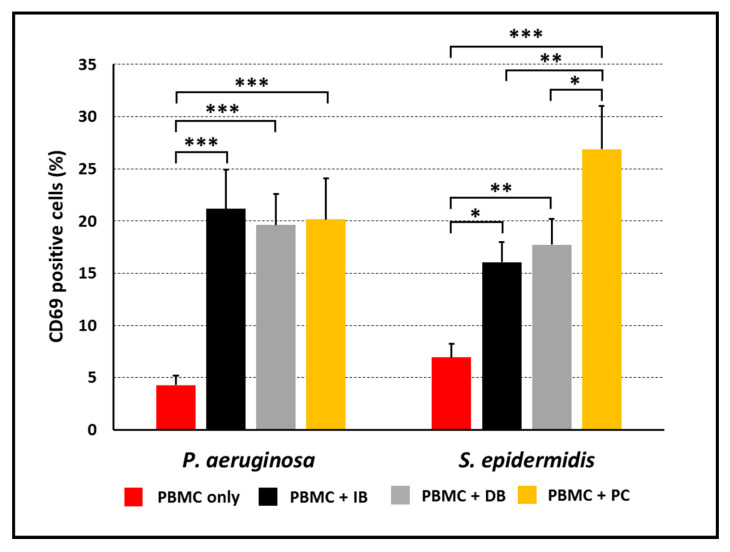
Expression of the early activation marker CD69 on PBMC stimulated for 24 h with intact biofilms (IB), disrupted biofilms (DB) or planktonic cells (PC). Unstimulated PBMC represented the negative controls. Mean values ± SEM from *n* = 7 (*P. aeruginosa*) and *n* = 8 (*S. epidermidis*) independent experiments are shown. * *p* < 0.05, ** *p* < 0.01, *** *p* < 0.001, ANOVA for matched samples followed by Bonferroni multiple comparisons test.

PBMC isolated from healthy blood donors were co-cultured with *P. aeruginosa* or *S. epidermidis* in planktonic or biofilm mode of growth for 24 h. In order to evaluate the possible influence on cell responses of the three-dimensional structure of intact biofilm (IB) as compared to planktonic bacteria (PC), a third experimental condition was included in the analysis for both species. This condition was represented by disrupted biofilms (DB) obtained by fragmenting 24 h-old biofilms via vigorous scraping and vortexing. DB were supposed to exhibit the same physiological state as bacterial cells within a mature biofilm, meanwhile being more accessible to host immune cells. The average percentages of different cell subsets in PBMC at time 0 is reported in Supplementary Figure S1. Following the stimulation period of 24 h, cells were harvested from the wells and their activation state was evaluated by flow cytometry. To this aim, the percentage of cells expressing the CD69 molecule, an early cell activation marker mainly expressed by activated T lymphocytes and NK cells was assessed. As shown in Figure 2, in all cases, stimulated PBMC expressed CD69 at statistically higher levels than PBMC incubated alone. Furthermore, while in response to *P. aeruginosa*, approximately 20% of the cells were activated with no major difference among IB, DB, and PC. In the case of *S. epidermidis*, the planktonic mode of growth induced a statistically higher degree of activation than both IB and DB. 

**Figure 3:**
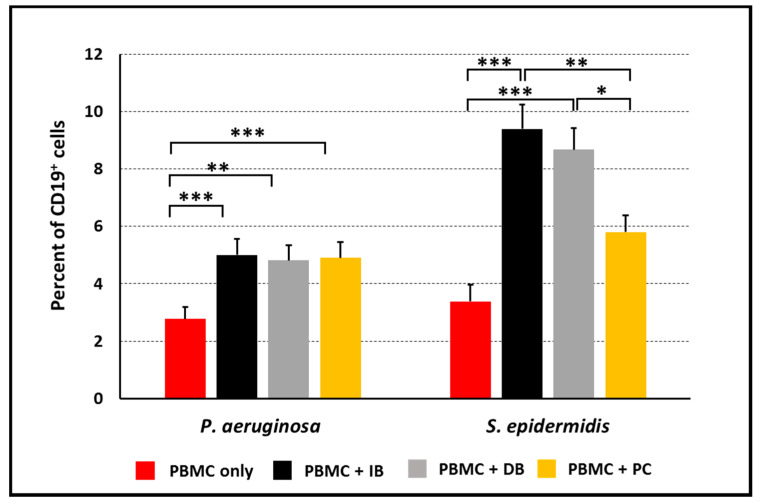
Percent of B lymphocytes among PBMC upon 24 h of incubation with intact biofilms (IB), disrupted biofilms (DB) or planktonic cells (PC). Mean values ± SEM from *n* = 7 (*P. aeruginosa*) and *n* = 8 (*S. epidermidis*) independent experiments are shown. * *p* < 0.05, ** *p* < 0.01, *** *p* < 0.001, ANOVA for matched samples followed by Bonferroni multiple comparisons test.

As CD69 marker is mainly expressed on T lymphocytes and NK cells; we sought to assess B cell activation by evaluating the expression of the HLA-DR marker on CD19^+^ cells. Although, HLA-DR is constitutively expressed on B cells, and its expression is reported to increase following activation [19]. No significant increase in the HLA-DR mean fluorescence intensity was observed on CD19^+^ cells upon co-culture with IB, DB, or PC of both bacterial species (data not shown). Nevertheless, the percentage of B cells raised in a statistically significant manner following stimulation with both *P. aeruginosa* and *S. epidermidis* (Figure 3). While in response to *P. aeruginosa* IB, DB, and PC, the percentages of B cells were similarly increased. IB and DB of *S. epidermidis* determined a higher increase in CD19^+^ cell-percentage as compared to planktonic cells, suggesting a biofilm-specific effect in the observed B cell-response. The increment in the percentage of CD19^+^ cells following stimulation was not due to a decrement in the number of other cell-subsets, as the same trend was confirmed calculating the absolute number of CD19^+^ cells in the different experimental conditions (Supplementary Table S1).

In all experimental conditions, <0.5% of the cells harvested after stimulation were CD14^+^ (monocytes), suggesting that they remained adhered to the wells, entrapped within the biofilm during the incubation period, or were lysed in the attempt to ingest bacterial cells.

### 3.3. Activated Cell-Subsets Following Stimulation with P. aeruginosa or S. epidermidis in Planktonic or Biofilm Mode of Growth

**Figure 4:**
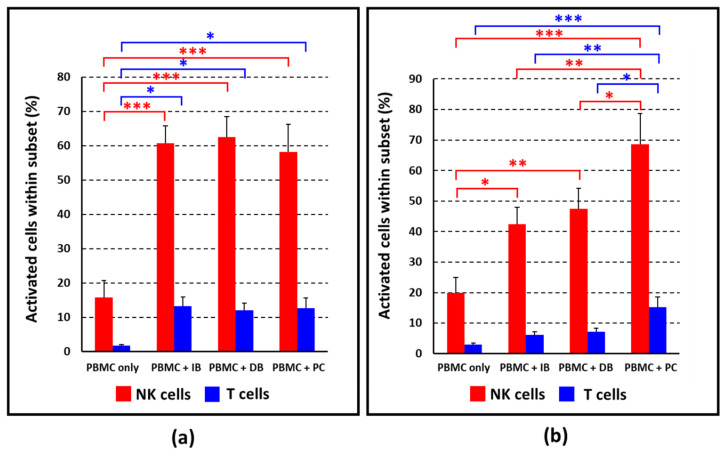
Activated cells (CD69+) among NK (CD56+/CD3-) cells and T (CD3+) cells. The percentage of activated (CD69+) cells within NK cell- and T cells, respectively, were calculated following 24 h of incubation of PBMC alone (PBMC only), with intact biofilm (PBMC + IB), disrupted biofilm (PBMC + DB), or planktonic cells (PBMC + PC) of *P. aeruginosa* (**a**) and *S. epidermidis* (**b**). Mean values ± SEM from *n* = 7 (*P. aeruginosa*) and *n* = 8 (*S. epidermidis*) independent experiments are shown. * *p* < 0.05, ** *p* < 0.01, *** *p* < 0.001, ANOVA for matched samples followed by Bonferroni multiple comparisons test.

The percentage of activated cells (CD69^+^) within T-cells and NK-cells was analyzed by flow cytometry following three-color fluorescent staining for the activation marker CD69 and the cell-surface markers identifying T cells (CD3) and NK cells (CD56), respectively. The results are shown in Figure 4 as mean values of the different donors tested and in Supplementary Figure S2 as a representative experiment. As shown in Figure 4a and Supplementary Figure S2a for *P. aeruginosa*, the vast majority of NK cells were activated in response to either IB, DB or PC with no evident difference among the three types of stimulants, while the percentage of CD69^+^ within T cells did not exceed 20%. In the case of *S. epidermidis* (Figure 4b and Supplementary Figure S2b), again, NK cells were those mostly activated in response to both biofilms and planktonic cells but with important differences depending on the stimulus. In particular, in response to PC, approximately 70% of NK cells were activated, while such percentages decreased in a statistically significant manner in response to DB and IB, respectively (Figure 4b). The percentage of activated cells within the T cell subset followed a similar trend.

### 3.4. Cytokine Secretion Profile Following Stimulation with P. aeruginosa or S. epidermidis in Planktonic or Biofilm Mode of Growth

**Figure 5:**
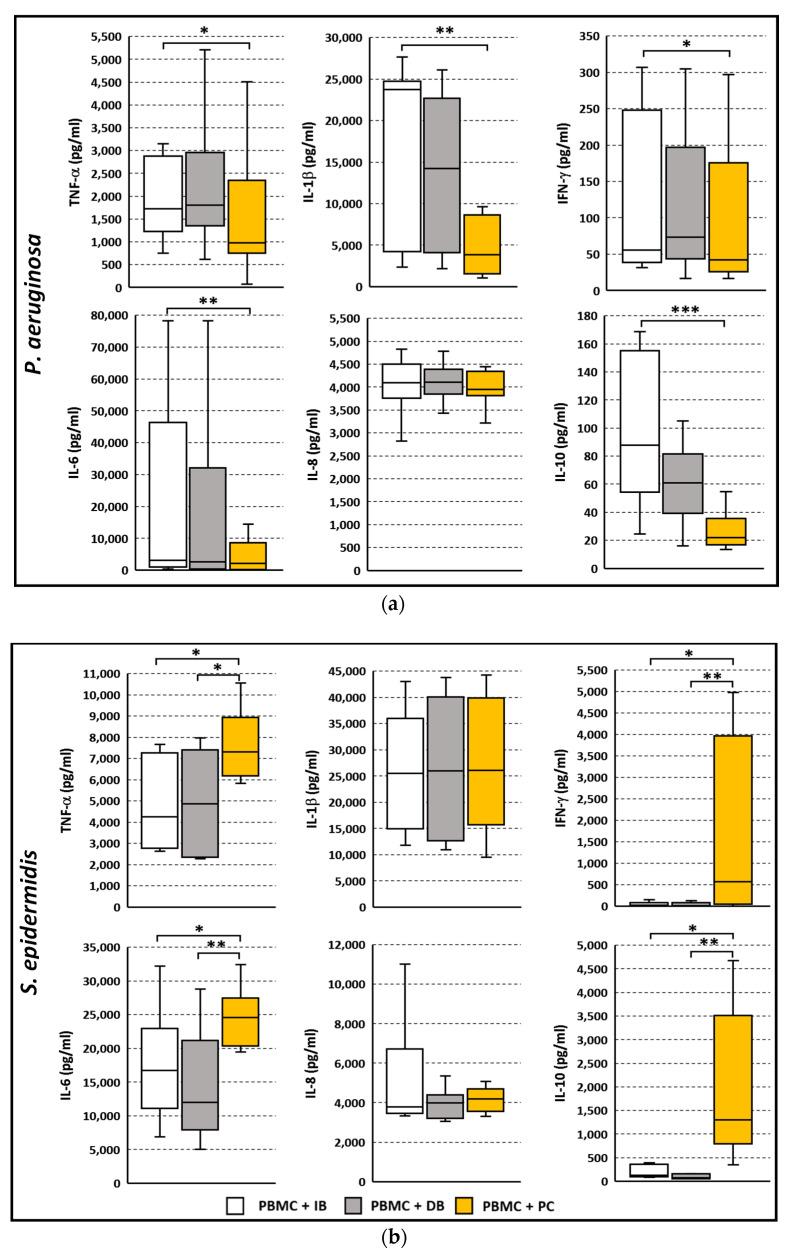
Cytokine profiles of PBMC co-cultured for 24 h with intact biofilms (IB), disrupted biofilms (DB), and planktonic cells (PC) of *P. aeruginosa* (**a**) or *S. epidermidis* (**b**). The supernatants were collected following 24 h stimulation and the cytokine amount was evaluated by flow cytometer based multi-bead capture assay. Data are presented as the median and interquartile range from *n* = 7 (*P. aeruginosa*) and *n* = 8 (*S. epidermidis*) independent experiments. * *p* < 0.05, ** *p* < 0.01, *** *p* < 0.001, non-parametric ANOVA for matched samples and Dunn’s multiple comparisons test.

The levels of the pro-inflammatory cytokines TNF-α, IL-1β, IFN-γ, IL-6 and IL-8, and of the anti-inflammatory cytokine IL-10 in the co-culture supernatants were assessed following 24 h stimulation with IB, DB, and PC. Despite *P. aeruginosa* IB, DB and PC elicited similar levels of activation in PBMC (Figure 2), a differential cell response to the three different bacterial stimuli was observed in terms of cytokine profiles. As shown in Figure 5a, *P. aeruginosa* IB induced statically higher levels of TNF-α, IL-1β, IFN-γ, IL-6, and IL-10 as compared to PC. Overall, the cytokine levels induced by DB were in between. IL-8 was induced at similar levels by *P. aeruginosa* IB, DB, and PC. In the case of *S. epidermidis*, a reverse trend was observed (Figure 5b). Statistically higher levels of TNF-α, IFN-γ, IL-6 and IL-10 were found in the supernatants of PBMC stimulated with *S. epidermidis* PC, as compared to cells stimulated with IB. Cytokine levels induced by IB and DB were similar. No significant difference was observed in the IL-1β and IL-8 production from PBMC co-cultured by three different bacterial stimuli. As expected, the cytokine levels of PBMC incubated alone were low or undetectable (data not shown).

### 3.5. Characterization of P. aeruginosa and S. epidermidis Biofilms in Terms of Biofilm-Architecture, Biofilm Biomass, and Biofilm-Associated Viable Bacteria Count

**Figure 6:**
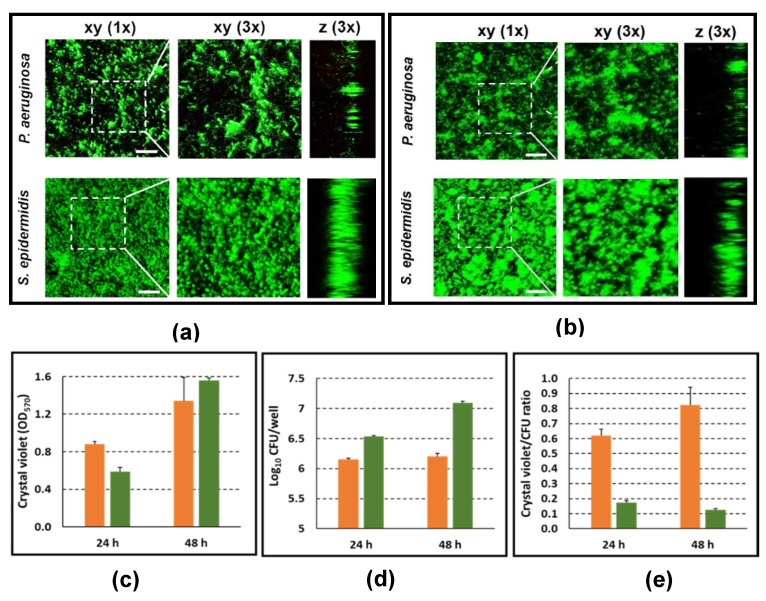
Characterization of *P. aeruginosa* and *S. epidermidis* biofilms. CLSM images of *P. aeruginosa* and *S. epidermidis* biofilms formed in complete RPMI 1640 medium at 24 (**a**) and 48 (**b**) h of incubation. Mature biofilms of *P. aeruginosa* and *S. epidermidis* were rinsed once to remove bacteria in the supernatant. After washing, biofilms were stained with green fluorescent labeled Syto9 (488/500–540 nm) (staining all the bacteria) and with red fluorescent propidium iodide (PI, 488/600–650 nm) (staining dead bacteria). Dashed square indicates the 3× zoomed area. Scale bar = 20 µm. In parallel wells, incubated in the same experimental conditions, the following were determined: total biomass of *P. aeruginosa* (green bars) and *S. epidermidis* (orange bars) biofilms by crystal violet (CV) staining (**c**); live biofilm-associated bacteria by CFU counting (**d**); and CV/CFU ratio by dividing the CV OD_570_ values to CFU counts (in million) (**e**). Mean values ± SEM from four wells for each species are shown from a representative experiment. PI data is not visible in the figure as for both species almost all bacteria within the biofilms were alive.

In an attempt to explain the differences observed in the activation levels and cytokine profiles of PBMC stimulated with biofilms and planktonic cells for each bacterial species, we proceeded to visualize the biofilm architecture at 24 h (time 0 of PBMC stimulation) and at 48 h (end of the stimulation period) by Live/Dead fluorescence staining and analysis by CLSM. In agreement with our previous study [15], in the adopted experimental conditions, *P. aeruginosa* formed biofilms consisting of characteristic micro-colonies separated by empty spaces, both at 24 and 48 h of culture (Figure 6a,b, respectively). In the same experimental conditions, *S. epidermidis* formed compact and thick biofilms with bacterial cells strictly adhered to each other (Figure 6a,b). Staining with propidium iodide revealed that, for both species, almost all of the bacterial cells within the biofilm were alive.

In order to quantify the total biofilm biomass and the biofilm associated viable bacteria count, in parallel wells, biofilms of both bacterial species were stained with CV, a dye known to stain both the bacterial cells and the extracellular matrix, or detached by vigorous scraping to assess the CFU count. As shown in Figure 6c, CV staining increased for both species during the 24 h incubation period. In the case of *P. aeruginosa*, such an increment was paralleled by a rise in the biofilm-associated viable count of approximately 0.7 Log (5 times), while the rise in the CFU count of *S. epidermidis* biofilms was less evident (Figure 6d). Both at 24 and 48 h, the CV/CFU ratio was higher for *S. epidermidis* than for *P. aeruginosa* (Figure 6e), suggesting that in *P. aeruginosa*, extracellular matrix contributes less to the total biofilm biomass than in *S. epidermidis*. 

### 3.6. Live Imaging of PBMC Interacting with P. aeruginosa and S. epidermidis Biofilms

**Figure 7:**
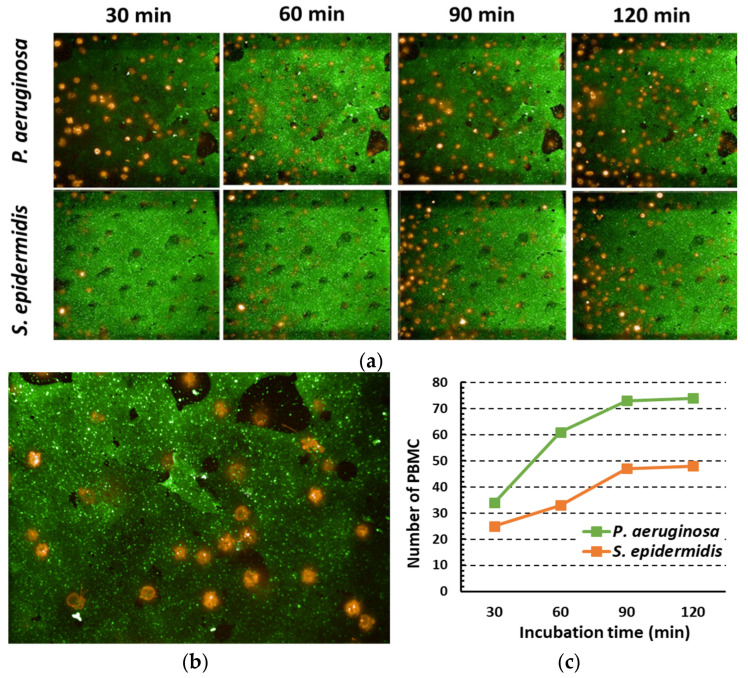
Live CLSM imaging of PBMC interacting with *P. aeruginosa* and *S. epidermidis* biofilms formed in complete RPMI 1640 medium. 24 h-old *P. aeruginosa* or *S. epidermidis* biofilms were labeled with 2 × 10^−6^ M green fluorescent lipophilic dye PKH67 (Merck), according to the manufacturer’s instructions, washed, and PBMC (2 × 10^6^ cells/mL, 4 × 10^5^ PBMC/well) prelabeled with 2 × 10^−6^ M orange/red lipophilic dye PKH26 (Merck) were added into the wells. Immediately after the addition of the cells, a time-lapse live imaging (nine plane confocal images at 63× magnification for each biofilm, every 5 min, for up to 150 min. at 37 °C with 5.5% CO_2_ atmosphere) of the co-cultures was performed. (**a**) PBMC infiltrating plane 5 at 30, 60, 90, and 120 min. for *P. aeruginosa* or *S. epidermidis* biofilms are shown from a representative experiment. (**b**) Further 4× digital magnification of PBMC infiltrating *P. aeruginosa* biofilm. (**c**) Number of PBMC infiltrating plane 5. Scale bar = 20 µm.

In order to further investigate the observed differences in the activation pattern and cytokine production in response to *P. aeruginosa* and *S. epidermidis*, live imaging experiments were conducted to visualize the interaction of biofilms with immune cells. To this aim, the Operetta confocal imaging system (Perking Elmer) of the Center for Instrument sharing (CISUP) of the University of Pisa was employed. Lipophilic fluorescent dyes were used to differentially stain bacterial (green) and immune cells (orange/red). Nine different planes were acquired on *Z*-axis in order to visualize the central part of the biofilm in depth (Supplementary Figure S3a). Figure 7 depicts the images of plane 5 obtained from a representative experiment. The biofilms of both species appeared as green layers with immune cells colored in orange/red infiltrating the biofilms (Figure 7a). Enlarged view (further 4x digital zoom) of *P. aeruginosa* biofilm (Figure 7b) shows that immune cells were localized mainly in the spaces between the macro-colonies. The computerized analysis demonstrated that, for both species, the number of immune cells interacting with the biofilms progressively increased in the first 30–90 min of co-culture and remained more or less constant afterward (Figure 7c). Quantification of immune cell numbers within each of the nine planes acquired is reported in Supplementary Figure S3b,c for *P. aeruginosa* and *S. epidermidis* biofilms, respectively.

Overall, this analysis demonstrated that the number of immune cells infiltrating the biofilm of *P. aeruginosa* was higher than that of *S. epidermidis* and suggested that the more compact and matrix-rich nature of the biofilms of the latter species may hinder the penetration of cells into the biofilm.

## 4. Discussion

It is widely recognized that the host immune response is only partially helpful in clearing biofilm-associated bacteria [14], but a detailed knowledge of the mechanisms involved and of the biofilm-specific properties, eliciting an inefficient immune response as compared to the planktonic life-style is only beginning to be understood. In this study, we sought to compare the human PBMC response to planktonic and biofilm forms of both *P. aeruginosa* and *S. epidermidis*, two clinically relevant bacterial species whose pathogenicity greatly relies on biofilm formation. For instance, *P. aeruginosa*’s ability to establish many chronic human infections such as chronic lung or wound infections is strictly dependent on biofilm formation that allows the bacterium to adapt to the host, retrieve nutrients and persist for decades in the tissues [20]. Conversely, *S. epidermidis*, regarded as an innocuous skin commensal for a long time, has become one of the most important causes of nosocomial infections in recent years, due its ability to form biofilms on indwelling medical devices, likely leading to bacterial invasion of blood circulation [21]. Studies comparing the immunological mechanisms stimulated by planktonic and biofilm types of bacterial growth have been previously undertaken for *S. aureus*, the species commonly considered the most pathogenic within the genus *Staphylococcus* [22,23,24], but less work has been dedicated to investigating such issues for *S. epidermidis*.

Many studies assessing the immune response to biofilms use, as a stimulant, bacterial cells mechanically dislodged from the biofilm [11,25]. This approach makes it simple to equalize the number of planktonic and biofilm bacteria but does not fully allow to take into consideration the possible influence of the three-dimensional biofilm structure on the response. For this reason, we included, in the comparison analysis, both disrupted (DB) and intact (IB) biofilms for *P. aeruginosa* and *S. epidermidis*. In theory, bacterial cells obtained by disrupting the biofilm reflect the same physiological state and antigenic composition of IB-cells, but they are more accessible to immune cells than those embedded in the biofilm. Conversely, bacteria growing in IB are enclosed in a complex and often species-specific 3D-structure that may influence the immune response acting as a barrier or as a source of antigenic stimuli.

To reliably compare the immune responses elicited by planktonic bacteria (PC) and bacteria embedded in the biofilm (IB), the first phase of the work was dedicated to standardizing bacterial numbers between these two conditions to ensure that eventual differences in the responses were not due to differences in the bacterial load between the two modes of growth. The standardized bacterial number also allowed attainment, at the end of the stimulation period, of an adequate number of live host cells that were comparable between PC and IB for both *S. epidermidis* and *P. aeruginosa*. 

Using this model, we first compared the levels of PBMC activation following 24 h co-culture with PC, IB and DB within each species. As for *P. aeruginosa*, in agreement with our previous study [15], IB were able to induce expression of the early activation marker CD69 on PBMC of different donors, but the levels of activation were similar to those induce by PC and DB. In contrast, in the case of *S. epidermidis*, a clear progressive increase in the expression of CD69 was observed passing from unstimulated cells to IB, DB, and PC respectively, suggesting that, in such species, the biofilm mode of growth may somehow hamper immune cells activation. 

Although HLA-DR, whose expression on CD19^+^ (B cells) is reported to increase following activation [19], was detected at similar levels on stimulated and non-stimulated B cells, both the percentage and the absolute number of B cells were increased, following stimulation with *P. aeruginosa* as well as *S. epidermidis*. Furthermore, in the case of *S. epidermidis*, but not of *P. aeruginosa*, IB and DB determined a more marked increase in the percentage of B cells than PC. Little is known about the role of B cells in the host response to biofilms of *S. epidermidis* as compared to planktonic cells [26]. Early studies in mouse spleen cells have demonstrated polyclonal B cell responses elicited by cell wall preparations of a number of Gram-positive species, including *S. epidermidis* [27]. A striking ability of B cells from human blood to associate with different strains of *S. epidermidis* was recently reported [28]. Our results suggest that specific components associated to or released by biofilms may preferentially interact with peripheral human B cells, but further studies are needed to clarify the nature of such interactions (polyclonal *versus* specific) and the putative components involved. The role of B cells and the humoral response needs to be better exploited in the case of *P. aeruginosa* infections. High numbers of B cells as well as serum levels of IgG2 and immune-complexes have been documented in CF patients chronically infected with *P. aeruginosa*, but their role is apparently ineffective if not directly harmful [29]. 

A striking finding of our study was the marked NK-cells (CD56^+^ CD3^−^) activation in response to both *P. aeruginosa* and *S. epidermidis*. In response to the former species, around 60% of NK cells were activated with no major difference among IB, DB and PC. These results confirm our previous observations that *P. aeruginosa* biofilm elicits a marked response of NK cells [15], and the results extend such observations not only to DB, but also to PC. Thus, NK cell activation does not seem to be biofilm-specific. Rather, it may be due to soluble factors released by accessory cells (e.g., monocytes) or T cells present in the culture, or to a direct recognition of bacterial components via NK cell receptors, a possibility demonstrated by others and us for different bacterial models [17,30,31]. Activation of the NK cell subset by *S. epidermidis* paralleled the trend observed in the total percentage of CD69^+^ cells in the co-cultures. Approximately 70% of NK cell were activated in response to *S. epidermidis* PC, and this percentage was statistically higher as compared to both IB and DB. To the best of our knowledge, NK cell activation in response to *S. epidermidis* has been rarely reported [32,33] and highlights a possible role of this innate immune cells in the host response to this pathogen.

To determine whether growth of bacteria in biofilms triggers specific effector functions, we compared the pattern of cytokine production by human PBMC stimulated with planktonic and biofilm bacteria (intact and dislodged). A different pattern of cytokine production was observed, depending on the lifestyle and the bacterial species. 

Despite of the fact that no difference was observed in CD69 expression following stimulation with the differentially growing *P. aeruginosa* (IB, DB and PC), higher levels of pro-inflammatory (TNF-α, IL-1β, IFN-γ, IL-6) and anti-inflammatory (IL-10) cytokines were elicited by IB than PC. Although the cytokine levels released in the supernatants upon stimulation with DB were not statistically different from those induced by IB (likely due to a high variability among donors), overall, such levels were in between IB and PC, suggesting that bacteria in intact biofilms exhibit specific features that are partially lost when they are dislodged from the 3D structure. The lack of correlation between expression of activation markers and cytokine-production observed for *P. aeruginosa* is currently difficult to explain, but it has been also found elsewhere, although in different bacterial models [34,35]. In our experimental systems, this might be due to the fact that at least part of the cytokines in the supernatant is likely to be released by adherent monocytes/macrophages (CD14^+^ cells). Although this cell type represented around 5–8% of PBMC at time 0, they represented less than 0.5% of the cells harvested following 24 h co-culture, suggesting that they adhered to the wells, remained entrapped within the biofilm, or lyzed during the incubation time. Our results are in agreement with a previous study reporting that *P. aeruginosa* growing in biofilm induces a higher production of TNF-α and IL-6 in human monocytes than its planktonic counterpart [11]. In the present study, the same trend was observed for other cytokines mainly produced by monocytes (e.g., IL-1β) by activated ΝΚ and T cell (e.g., IFN-γ) or by both (e.g., IL-10). Several factors could explain the enhanced cytokine response of *P. aeruginosa* biofilms as compared to planktonic cells. For instance, Ciornei et al., demonstrated that once *P. aeruginosa* switches from the planktonic to the biofilm mode of growth, its lipopolysaccharide undergoes a number of structural modifications regarding both the lipid A and the polysaccharide moieties [11]. Such a switch occurs in both clinical isolates and laboratory strains [11]. In in the same study, the authors also demonstrate that LPS isolated from biofilm-growing bacteria induces slightly more inflammatory cytokines than LPS extracted from their planktonic counterpart explaining, at least in part, the increased inflammatory response elicited by biofilms. Another explanation may be that the existence of biofilm-specific components (e.g., extracellular DNA, other EPS components, proteins up-regulated in biofilms) are not only able to activate, but also able to induce an enhanced cell response as compared by antigenic determinants associated to planktonic cells. 

As for IFN-γ, an unusual production pathway of this cytokine associated with cell death in macrophages infected with different bacterial pathogens has been previously described [36]. This finding may suggest that monocytes dying in an attempt to engulf *P. aeruginosa* biofilm cells, which exhibit greater resistance to phagocytosis as compared to their planktonic counterparts [37], may represent a strong stimulus for IFN-γ production, providing another explanation for the differential release of this cytokine between biofilm and planktonic cells. 

In the case of *S. epidermidis,* an overall opposite trend of that just discussed for *P. aeruginosa* was observed. For this bacterial species, PC tended to induce higher levels of cytokine release (e.g., TNF-α, IFN-γ, IL-6 and IL-10) than IB or DB. IFN-γ and IL-10, in particular, were almost exclusively produced in response to PC. Biofilm formation is considered the primary virulence factor of *S. epidermidis*, which uses such a mode of growth to escape both antimicrobial therapy and the host immune system [38]. It has been reported that *S. epidermidis* biofilms hamper IgG and complement deposition, resulting in a high resistance to opsonization and phagocyte-mediated killing [39,40]. Furthermore, *S. epidermidis* biofilms have been reported to contain higher amounts of dormant cells and to induce lower production of TNF-α, IL-12 and IL-6 than planktonic cells, in murine bone marrow-derived dendritic cells in vitro [35]. In another study, addressing human PBMC responses, biofilm phase *S. epidermidis* elicited lower amounts of pro-inflammatory cytokines such as TNF-α, IL-12p40, IL-12p70 and IFN-γ, than planktonic phase bacteria, but a higher amount of IL-8, GM-CSF and IL-13 [24]. Altogether, the results obtained in the present and previous studies point to a low pro-inflammatory profile of *S. epidermidis* biofilms.

A major component of *S. epidermidis* biofilms is the EPS. One of the best characterized components of *S. epidermidis* EPS is PIA, a linear homopolymer of β-1,6-linked N-acetylglucosamine monomers partially deacetylated and encoded by the *icaADBC* operon [41]. A major role of PIA in evasion mechanisms of *S. epidermidis* biofilms is supported by ex vivo and in vivo studies [42]. For instance, when tested in a human whole blood ex vivo model of infection, a PIA-positive *S. epidermidis* strain (SE1457) induced lower levels of TNF-α, IL-6, IFN-γ production than its PIA-negative isogenic mutant (M10) [43]. In a mouse model of catheter-associated infection, more virulent wild-type *S. epidermidis* was killed more slowly by polymorphonuclear cells than an *ica*-negative strain in an ex vivo killing assay [39]. The strain used in the present study is a strong producer of PIA [10] whose expression is markedly more pronounced on biofilm cells than planktonic cells [25]. Thus, the biofilm-extracellular matrix may have masked the antigenic determinants and represented a physical barrier to the PBMC-biofilm interaction, leading to the low cell-activation and cytokine production observed in this study in response to *S. epidermidis* IB as compared to PC. This hypothesis is sustained by the confocal microscopy data that showed a thicker and more compact structure of *S. epidermidis* biofilm as compared to *P. aeruginosa* biofilms and a higher matrix content per cell (crystal violet/CFU ratio) in the former species than in the latter. Time-lapse confocal analyses demonstrated the penetration of a higher number of PBMC into the *P. aeruginosa* biofilms as compared to the *S. epidermidis* biofilms to further strengthen this explanation.

**Figure 8:**
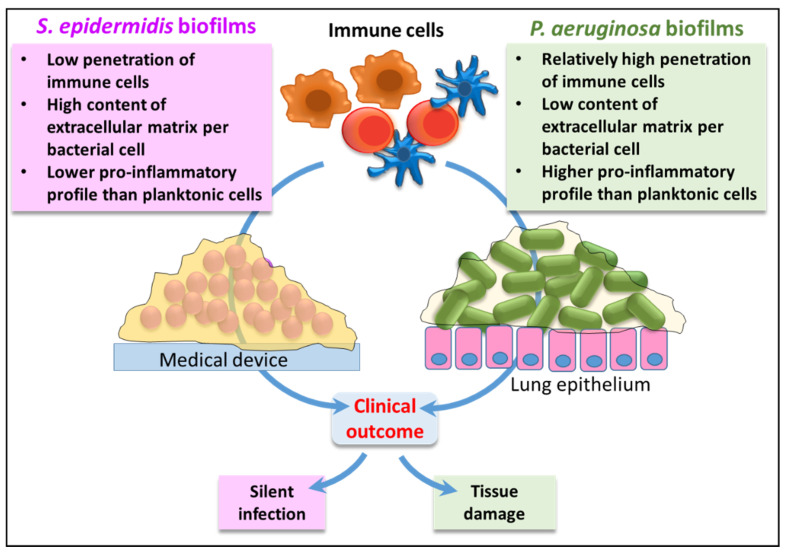
Hypothesis based on the results of the present study on the different strategies adopted by *S. epidermidis* and *P. aeruginosa* biofilms to establish long lasting relationship with the host and possible clinical outcomes.

We are aware that our study handles an in vitro biofilm model. The in vivo situation is probably far more complex in which many other factors may play a role. Nevertheless, the results obtained suggest different strategies adopted by *P. aeruginosa* and *S. epidermidis* biofilms to persist in the host (Figure 8).

To adapt to hostile environments, such as CF lung, *P. aeruginosa* undergoes a plethora of evolutionary changes that include the switch to the biofilm mode growth, loss of motility and reduced virulence factor production [44]. The bacterium can thus compensate this loss of virulence by changing its antigen repertoire (e.g., LPS) and inducing a stronger inflammatory response as compared to planktonic cells with consequent collateral tissue damage that it can probably exploit to obtain nutrients and spread in the surrounding tissues. Furthermore, cytokines released by innate immune cells or T cells have been demonstrated to bind bacterial cells [45] and stimulate bacterial growth [46], release of bacterial components (e.g., rhamnolipids) [47], or biofilm formation [15,48], suggesting that *P. aeruginosa* may “sense” the presence of host immune components and actively react to promote its own persistence. 

Conversely, the ability of *S. epidermidis* in the biofilm mode of growth to avoid recognition by immune cells and hamper their activation while maintaining a low inflammatory profile may be the strategy preferred by this species to establish silent and long-lasting relationship with the host, persisting on the surface on implanted devices [38]. 

## 5. Conclusions

In conclusion, the results obtained in this study disclose complex interplays between two relevant bacterial species and the host immune cells. The outcomes of these interplay may vary according to the lifestyle (biofilm versus planktonic) and the bacteria species. In particular, *P. aeruginosa* biofilms revealed to be more potent inducers of pro- and anti- inflammatory cytokines as compared to planktonic cells, while for *S. epidermidis*, it was the opposite. The results obtained also highlighted possible roles played by poorly investigated cell subsets in the human immune response to biofilms or planktonic cells (e.g., NK cells), and overall, contributed to our understanding of the mechanism(s) causing persistence of chronic bacterial infections. 

## Data Availability

The datasets generated for this study are available on request to the corresponding author.

## Supplementary Materials

The following are available online at https://www.mdpi.com/article/10.3390/microorganisms9091846/s1. Scheme S1: Main steps of the experimental procedure adopted to setup the bacteria: PBMC co-cultures; Figure S1: Percentages of different cell subsets in PBMC subjected to stimulation with *P. aeruginosa* or *S. epidermidis* (biofilms, disrupted biofilms or planktonic bacteria) at time 0; Figure S2: Percentages of activated cell subsets (T cells, NK cells, and B cells) in PBMC subjected to stimulation with *P. aeruginosa* or *S. epidermidis*; Figure S3: Computer assisted quantification of immune cell (PBMC) numbers interacting at different times and at different depths with the biofilm of *P. aeruginosa* and *S. epidermidis*; Table S1: Absolute cell numbers of PBMC subsets after 24 h culture with or without bacteria.
