# Supplementary material for: Planktonic and Biofilm-Associated Pseudomonas aeruginosa and Staphylococcus epidermidis Elicit Differential Human Peripheral Blood Cell Responses
Source: Microorganisms. 2021 Aug 31;9(9):1846. doi: 10.3390/microorganisms9091846 (PMC8470397; doi:10.3390/microorganisms9091846)
Supplement: Supplementary file 1 [file microorganisms-09-01846-s001.zip › microorganisms-1338178-supplementary.pdf]

Response of human peripheral blood mononuclear cells to *Pseudomonas aeruginosa* and *Staphylococcus epidermidis* in planktonic and biofilm mode of growth

Esingul Kaya[1†], Giovanna Batoni[1†], Maria Grazia di Luca[2], Eleonora Apolloni[1], Alessandro Mazzoni[3], Giuseppantonio Maisetta[1], and Semih Esin[1*]

Supplementary materials

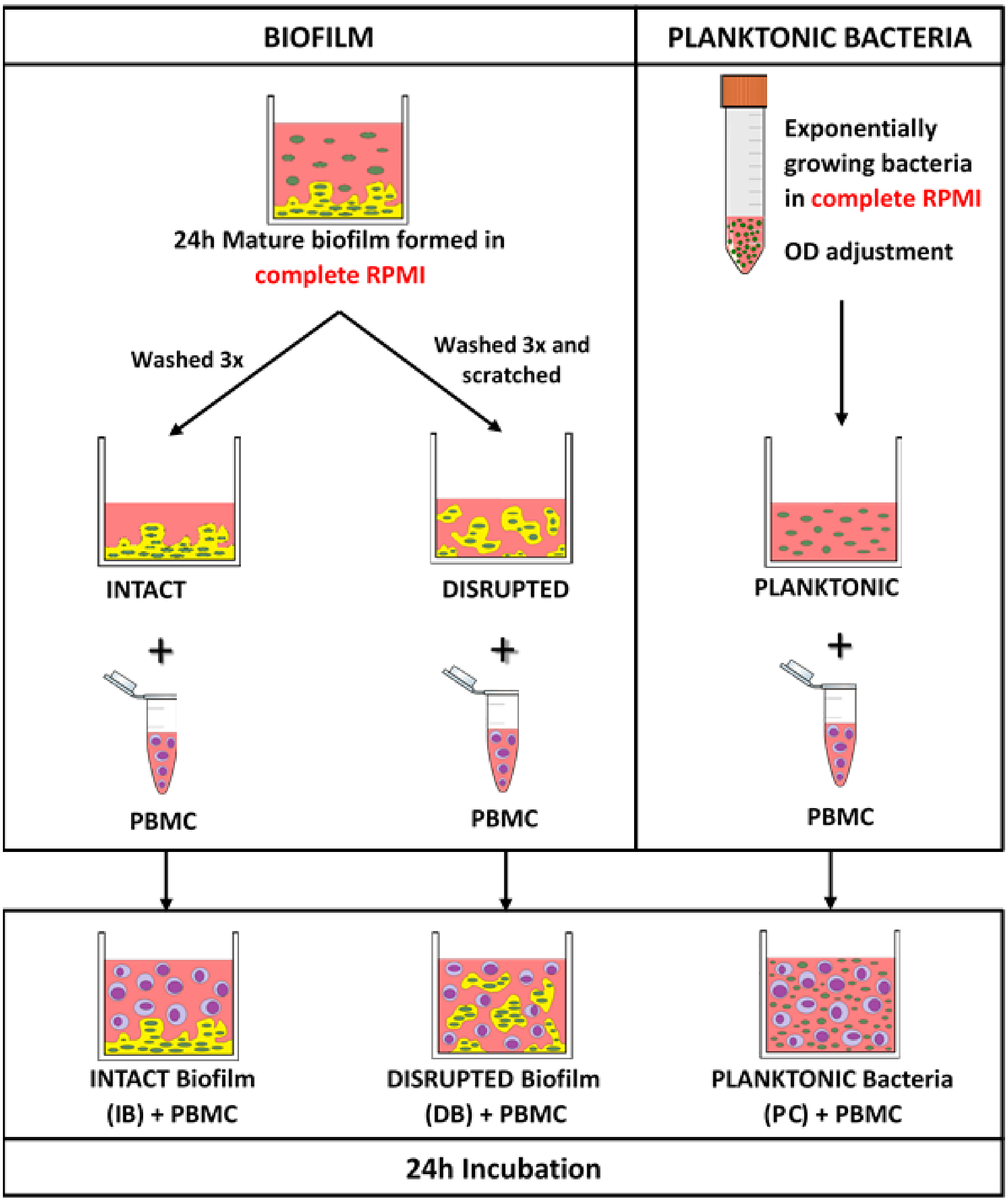

Scheme S1. Main steps of the experimental procedure adopted to setup the bacteria:PBMC co-cultures.

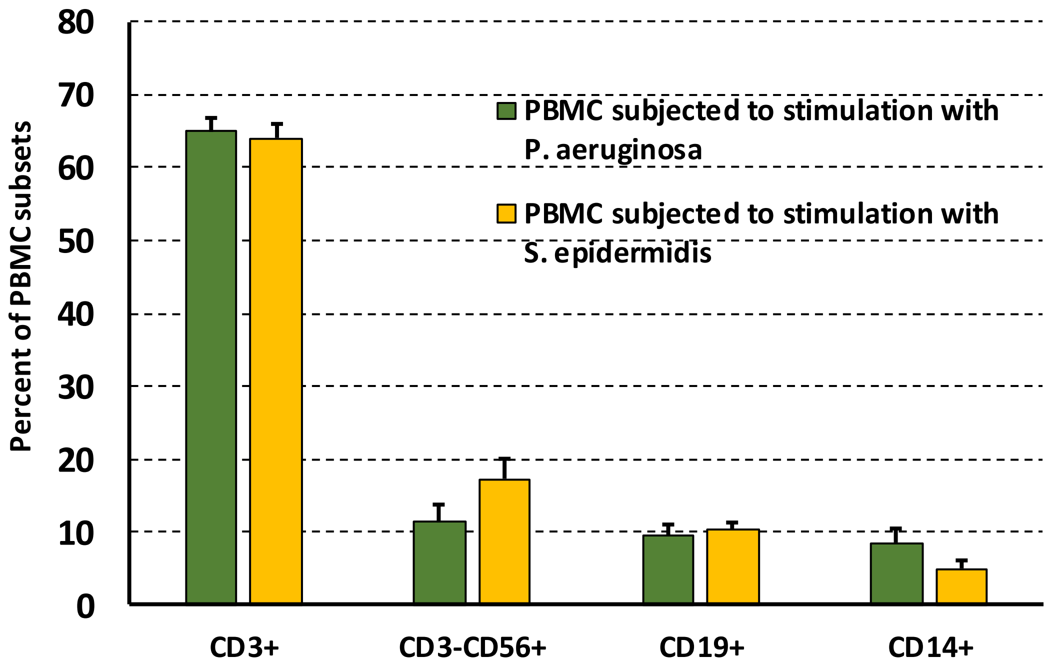

Figure S1. Percentages of different cell subsets in PBMC subjected to stimulation with *P. aeruginosa* or *S. epidermidis* (biofilms, disrupted biofilms or planktonic bacteria) at time 0. The figure shows the mean values ± SEM of the 8 donors tested for *P. aeruginosa* and of the 7 donors tested for *S. epidermidis*. Two donors were tested with both species.

Table S1. Absolute cell numbers of PBMC subsets after 24h culture with or without bacteria.

	P. aeruginosa			*S. epidermidis*		
	**T cells**	**NK cells**	**B cells**	**T cells**	**NK cells**	**B cells**
**PBMC**	252909±44803	17234±3688	8915±2148	184647±12394	34822±13440	9354±1865
**IB**	190964±33139	15490±2581	11532±2029	180516±29854	50134±23259	23144±4647
**DB**	202051±38697	13895±1933	11640±2266	176903±28885	50737±19656	20983±3847
**PC**	177837±40275	11386±1967	11039±2353	176907±25588	45224±18546	13849±1989

Mean values ± SEM from n=7 (*P. aeruginosa*) and n=8 (*S. epidermidis*) independent experiments are shown. Numbers of monocytes were not shown as they were less than 0.5 % in all conditions.

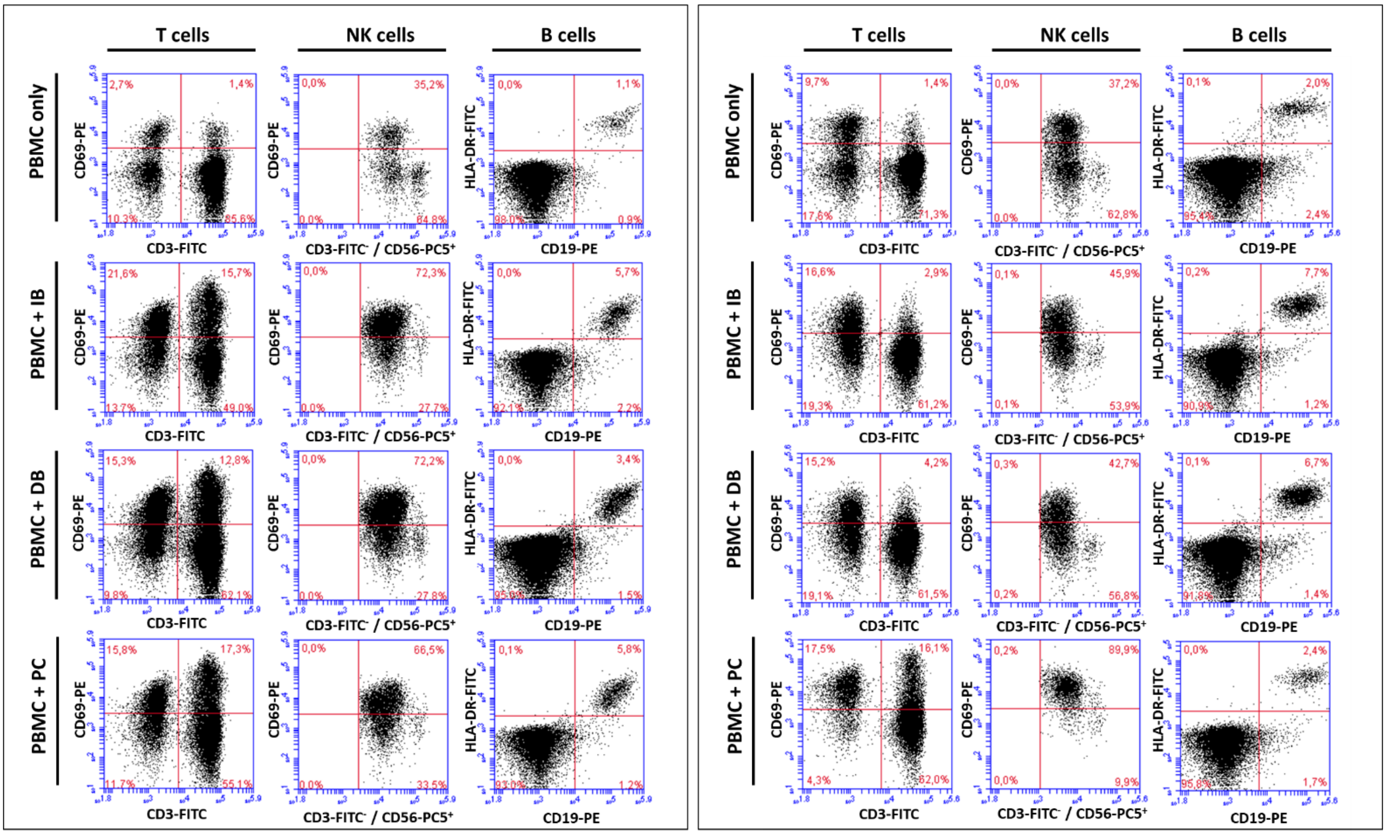

(a) **(b)**

Figure S2. Percentages of activated cell subsets (T cells, NK cells, and B cells) in PBMC subjected to stimulation with *P. aeruginosa* or *S. epidermidis*. The figure shows data (dot plots) of PBMC from a representative donor tested for *P. aeruginosa* (**a**) and for *S. epidermidis* (**b**). IB: intact biofilms; DB: disrupted biofilms; PC: planktonic bacteria. For NK cells CD69 staining on $CD56^{+}CD3^{-}$ cells gated from CD3 *versus* CD56 plots are shown.

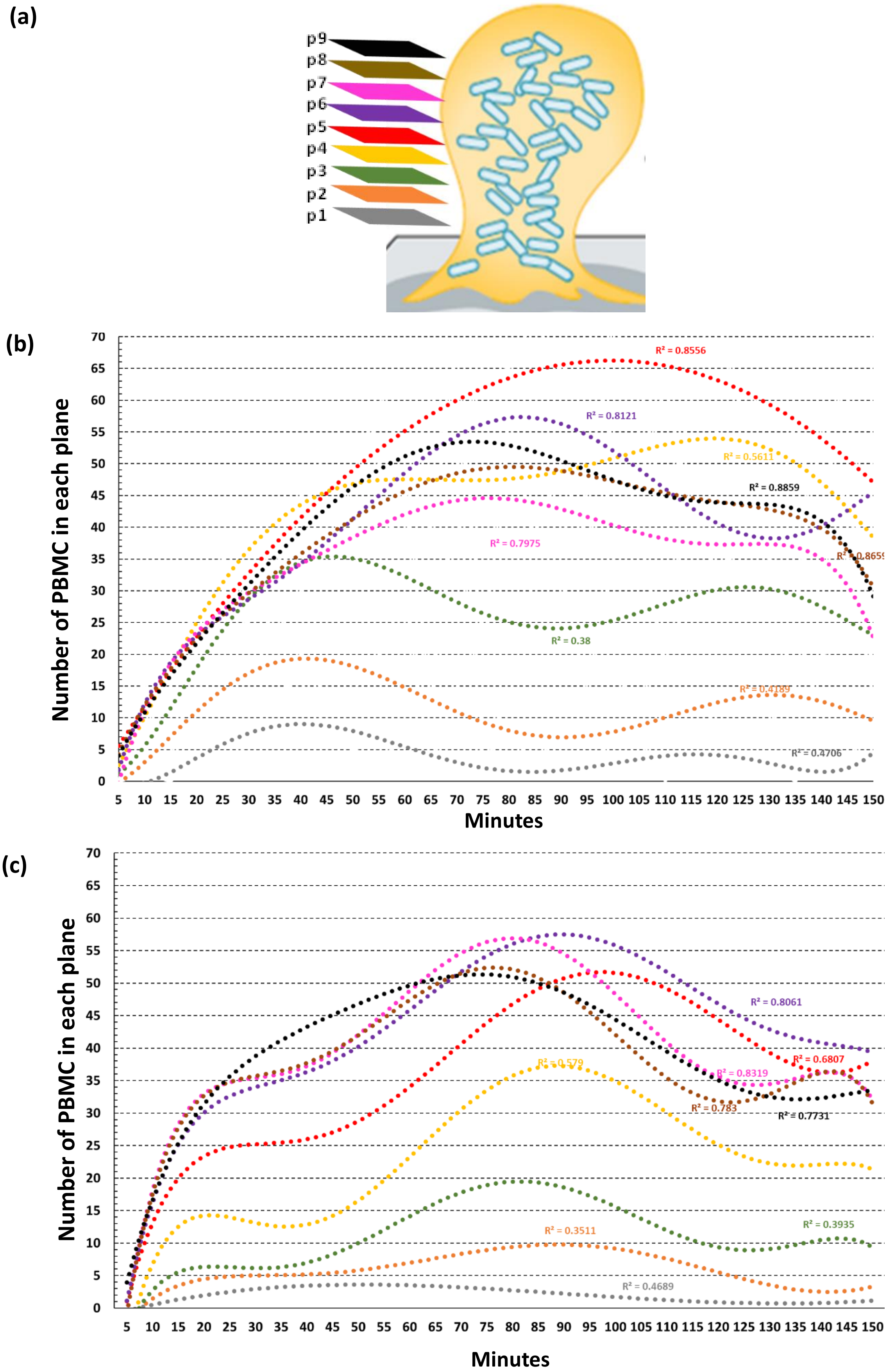

Figure S3. Computer assisted quantification of immune cell (PBMC) numbers interacting at different times and at different depths with the biofilm of *P. aeruginosa* and *S. epidermidis*. Images from nine different planes, from p1 (the deepest) to p9 (the outermost) were acquired for biofilms of both species (**a**). Immune cell numbers were evaluated in each plane via Harmony software (Perkin Elmer Inc.) for *P. aeruginosa* (**b**) and *S. epidermidis* (**c**). Graphics depict the trend-lines elaborated according to polynomial equation by Microsoft Excel. In the case of *P. aeruginosa*, most of the cells were localized in the middle section of the biofilm (i.e., plane 5) after 60-90 min. of co-incubation, whereas in the case of *S. epidermidis*, the majority of the cells were found in superficial planes (i.e., 6, 7, 8, and 9). Few cells were able to reach the deepest layers of the biofilms. R^2: R-squared value for reliability of each trend-line.
